# Endothelial Notch signaling directly regulates the small GTPase *RND1* to facilitate Notch suppression of endothelial migration

**DOI:** 10.1038/s41598-022-05666-1

**Published:** 2022-01-31

**Authors:** Bhairavi Swaminathan, Seock-Won Youn, L. A. Naiche, Jing Du, Stephanie R. Villa, Jordan B. Metz, Huijuan Feng, Chaolin Zhang, Raphael Kopan, Peter A. Sims, Jan K. Kitajewski

**Affiliations:** 1 Department of Physiology and Biophysics, University of Illinois Chicago, Chicago, IL 60612 USA; 2 Department of Systems Biology, Department of Biochemistry and Molecular Biophysics, Columbia University, New York, NY 10032 USA; 3 Division of Developmental Biology, Department of Pediatrics, University of Cincinnati College of Medicine and Cincinnati Children’s Hospital Medical Center, Cincinnati, OH USA

**Keywords:** Cardiovascular biology, Cell migration, Cell signalling, Gene regulation, Angiogenesis

## Abstract

To control sprouting angiogenesis, endothelial Notch signaling suppresses tip cell formation, migration, and proliferation while promoting barrier formation. Each of these responses may be regulated by distinct Notch-regulated effectors. Notch activity is highly dynamic in sprouting endothelial cells, while constitutive Notch signaling drives homeostatic endothelial polarization, indicating the need for both rapid and constitutive Notch targets. In contrast to previous screens that focus on genes regulated by constitutively active Notch, we characterized the dynamic response to Notch. We examined transcriptional changes from 1.5 to 6 h after Notch signal activation via ligand-specific or EGTA induction in cultured primary human endothelial cells and neonatal mouse brain. In each combination of endothelial type and Notch manipulation, transcriptomic analysis identified distinct but overlapping sets of rapidly regulated genes and revealed many novel Notch target genes. Among the novel Notch-regulated signaling pathways identified were effectors in GPCR signaling, notably, the constitutively active GTPase *RND1*. In endothelial cells, *RND1* was shown to be a novel direct Notch transcriptional target and required for Notch control of sprouting angiogenesis, endothelial migration, and Ras activity. We conclude that *RND1* is directly regulated by endothelial Notch signaling in a rapid fashion in order to suppress endothelial migration.

## Introduction

Notch signaling regulates angiogenesis by controlling diverse endothelial cell behaviors, including migration, proliferation, metabolism, sprout formation, and barrier integrity. To control sprouting angiogenesis, Notch signaling must integrate these cellular steps in a temporal and context dependent manner^1^. While Notch acts as a receptor at the cell surface, activation by Notch ligands from the Delta like (*DLL*) or Jagged (*JAG*) families permit γ-secretase cleavage^2,3^ of the Notch Intracellular domain (ICD) which then translocates to the nucleus, forms a transcriptional complex with CSL/RBP-J and MAML, and drives transcription of genes containing RBP-J binding sites^4,5^. In endothelial cells, Notch transcriptional targets include those that are directly regulated, such as VEGFR3/*FLT4*^6^, or those that respond to further transcriptional regulation, such as the transcriptional repressors Hairy and Enhancer-of-split family—*HES* and *HEY* genes^1,7–9^. Angiogenic control by Notch is driven by gene transcription and of both direct and secondary targets that control cellular behaviors, termed Notch effectors. Many Notch targets have been identified, but only in select cases has an effector function been delineated*,* for example *NRARP* controls vessel regression^10,11^ and EFNB2 promotes migration and proliferation^12,13^.

Genome-wide screens for Notch/CSL binding sites or direct Notch targets have been conducted in a variety of immortalized cell types, including T-lymphoblastic leukemia cells, myoblasts, and mouse kidney cells^14–21^. These screens have demonstrated that Notch targets vary widely by cell type, suggesting that targets identified in immortalized cells are not broadly applicable in endothelial cells where, for instance, Notch regulates the VEGF Receptor genes (VEGFR1, VEGFR2, and VEGFR3). Prior screens to identify Notch targets have generally utilized constitutive signaling via activating mutations or overexpression of Notch ICD. Paradoxically, examination of genes regulated by constitutive Notch ICD expression may not optimally detect direct Notch targets, due to rapid upregulation of transcriptional repressors of the Notch pathway^1,7–9^. In studies conducted in primary endothelial cells, microarray analysis of constitutively activated Notch signaling have identified downstream genes, such as *VEGFR1*, but have not been able to capture the range of rapidly regulated genes and did not distinguish between direct and indirect targets^22,23^.

We hypothesized that identification of genes rapidly regulated by endothelial Notch signaling in a timeframe similar to that of established direct targets would indicate novel direct Notch targets and reveal novel Notch effectors that function in angiogenesis. We therefore profiled transcriptional changes in EC in response to Notch induction and Notch repression in vitro and in vivo focusing on transcriptional events that happened within 1–6 h, rather than long-term changes in response to constitutive Notch signaling. We defined rapidly regulated Notch targets in complementary assays, including ligand-specific induction with tethered Delta-like ligand (DLL), Notch activation with EGTA, Notch inhibition by GSI in vitro and in vivo. Overlapping profiles from these different screens identified numerous previously unreported Notch regulated genes and an unappreciated mechanism of Notch signaling via GPCR and small GTPase proteins. Particularly interesting was *RND1*, a constitutively active Rho GTPase that is rapidly regulated by Notch, is a direct transcriptional target, controls endothelial Ras activity, and facilitates the control of endothelial migration by Notch.

## Results

### DLL4-activated and gamma-secretase dependent expression profiles identify endothelial Notch-regulated genes

**Figure 1:**
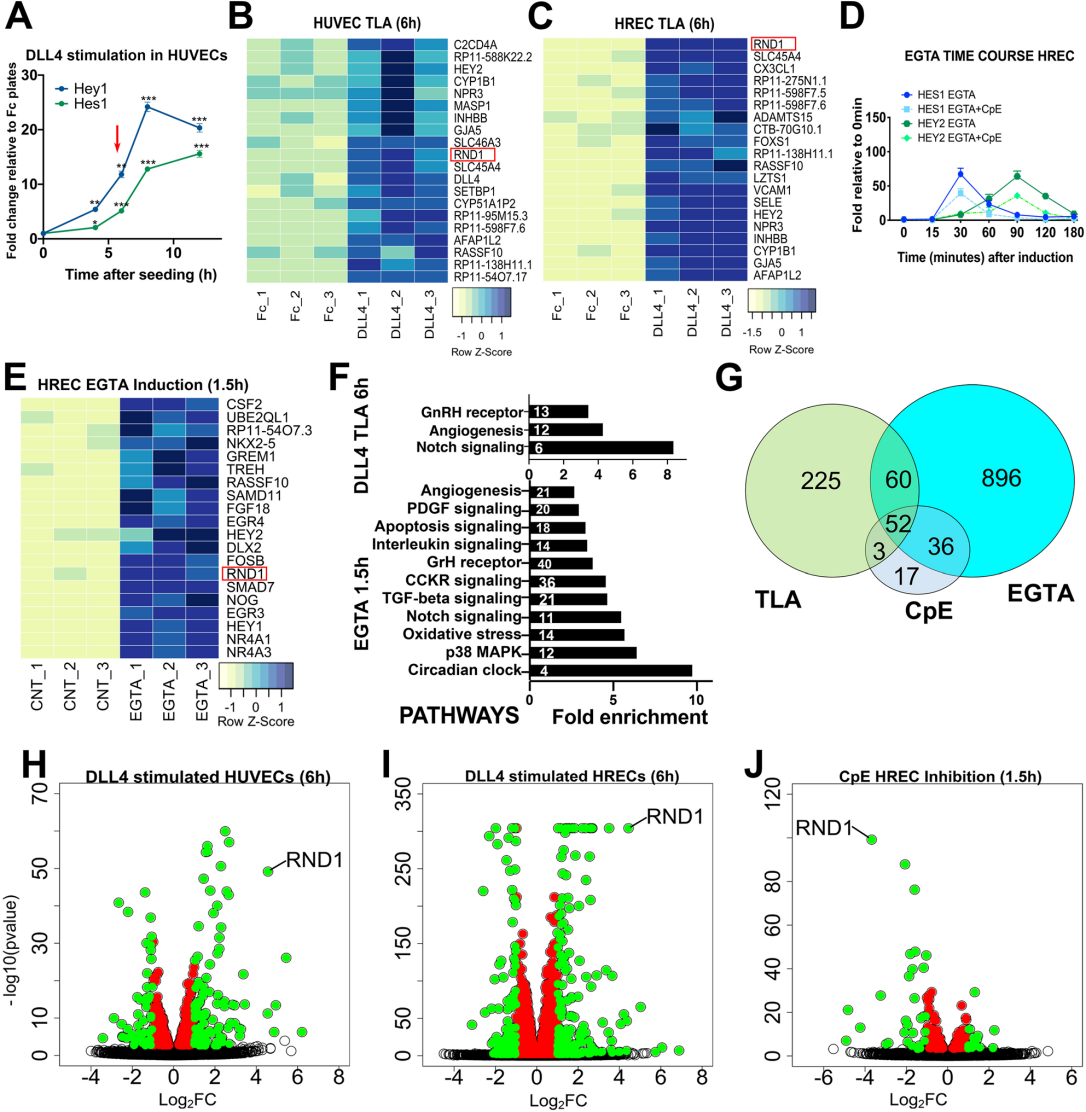
Ligand Notch activation rapidly induces known and novel targets including *RND1* in endothelial cells. (**A**) Time course of induction after seeding HUVEC onto DLL4-Fc coated plates as compared to Fc coated control plates (TLA assay). Robust activation of expression of known direct Notch targets was achieved as early as 6 h (red arrowhead). Single, double, and triple asterisks indicate *p* values of < 0.05 (*), < 0.01 (**), and < 0.001 (***), respectively, in all panels. (**B–C**) The 20 genes with the highest average fold induction in HUVEC (**B**) or HREC (C) by DLL4 TLA after 6 h. All heatmaps indicate z-score (standard deviations from the mean). *RND1* is among the most significantly regulated Notch targets in both EC cell types. Red rectangle indicates *RND1* in all heatmaps. (**D**) Induction time course of known Notch targets in HREC after 15 min of EGTA treatment or treatment with both EGTA and CpE. Robust activation of known direct Notch targets occurs between 30 and 90 min (0.5–1.5 h). (**E**) Heatmap of the 20 genes most highly upregulated by EGTA induction in HREC within 1.5 h shows that *RND1* is rapidly significantly upregulated within the same timeframe as known direct Notch targets. (**F**) GO pathways significantly enriched in the 340 genes significantly upregulated by DLL4 TLA induction in both HUVEC and HREC (top) or the 1,105 genes significantly upregulated by EGTA (bottom). Inset indicates number of significantly regulated genes contributing to each pathway. The Notch signaling pathway was significantly regulated in both contexts. (**G**) Overlap between genes significantly upregulated by DLL4 TLA induction in both HUVEC and HREC, genes induced by EGTA, and genes repressed by CpE. (**H–J**) Volcano plots of gene expression changes in DLL4-stimulated HUVEC, DLL4-stimulated HREC, and CpE-inhibited EGTA-stimulated HREC (left to right, respectively). Uncolored circles indicate p_adj_ > 0.05, colored circles indicate p_adj_ < 0.05, red circles = fold change (FC) <|1.2|, green circles = FC >|1.2|.

To characterize ligand-specific and rapid transcriptional changes downstream of Notch, we tethered the extracellular domain of recombinant Notch ligand DLL4 to plates on which we subsequently seeded ECs to perform a Tethered Ligand Assay (TLA) as previously described^24^. This approach allowed us to control the time of onset of Notch signaling (i.e. when the cells settle onto the ligand) and avoids super-physiologic and ligand non-specific activity caused by NICD^25^. In optimizing this assay, we documented that known Notch direct targets, such Hey1 and Hes1, could be consistently regulated with high significance at 6 h. We have therefore defined “rapidly-regulated” genes throughout this study as those which can be detected when known direct Notch targets are robustly induced. Human Umbilical Vein ECs (HUVECs) stimulated by tethered 10 ug/ml DLL4 showed robust activation of Notch canonical targets 6 h after seeding (Fig. 1A).

To define the DLL4-regulated transcriptome, we selected two complementary primary endothelial cell types, HUVEC and Human Retinal ECs (HREC) (Fig. S1). HUVEC are proliferative, highly angiogenic, and the standard EC type for most assays of sprouting angiogenesis. Recent consensus among investigators studying angiogenesis suggests that results should be confirmed with a microvascular EC type^26^. We selected HREC as a microvascular EC of the central nervous system that is highly relevant to retinal assays of angiogenesis. We conducted triplicate DLL4-TLA assays with either HUVEC from two donor pools or HREC from a single donor, isolated RNA 6 h after cell seeding, and sequenced at a depth of 30 M single-end (SE) or 30 M paired-end (PE) reads for HUVEC and HREC, respectively. The complete RNA sequencing dataset is available in the NCBI Gene Expression Omnibus repository at https://www.ncbi.nlm.nih.gov/geo (Accession number GSE163568). DLL4-mediated Notch activation resulted in significant (p_adj_ < 0.05) changes in expression of hundreds of genes in both HUVEC (394 genes upregulated, 326 genes down-regulated) and HREC (1643 genes upregulated, 1701 genes downregulated) (Fig. 1B,C, S2A-B). 340 genes were upregulated at least 1.2 fold (Log_2_FC > 0.27) in both EC cell types. DLL4 TLA strongly upregulated known Notch targets *HEY2, GJA5, and GUCY1A1* and GO pathways “Notch Signaling” and Angiogenesis”, consistent with Notch activation (Fig. 1F), but many of the most strongly regulated genes were not previously known to be Notch targets (Fig. 1B,C and Fig. S2G).

To detect transcriptional responses more rapidly than possible in the TLA assay and thus enrich for Notch direct targets, we screened for endothelial Notch targets using brief (15 min) exposure of HREC to EGTA, which promotes S2 cleavage of Notch within minutes of addition. Upregulation of known Notch targets *HES1* and *HEY2* were observed 30–90 min after HREC were treated with 10 mM EGTA and significantly repressed by 500 nM of the GSI Compound E (CpE) (Fig. 1D). This precise temporal activation of Notch permitted measurable responses within minutes and up to 2 h after Notch activation. Transcriptional profiling showed that EGTA treatment significantly upregulated 1105 genes by at least 1.2-fold in HREC after 1.5 h (Fig. 1E and S2C). To minimize conflation with other EGTA-activated pathways, we compared the transcriptional profile of EGTA-activated HREC to those pretreated with CpE to block S3 cleavage and inhibit Notch activation. 88 of the activated genes were significantly repressed by CpE (Fig. S2D-E).

Comparing transcriptional signatures from DLL4-mediated and gamma-secretase dependent Notch activation in two EC cell types identified a set of 52 commonly regulated genes likely to be direct Notch targets in endothelial cells (Fig. 1G). These included canonical Notch targets and a 55-fold enrichment for the Notch pathway signature (Fig. S2F-G). We also identified candidate Notch effectors not previously known to interact with Notch signaling, including small GTPases *RND1* and *RAPGEF5*, and Ras-associated domain Family protein (*RASSF10*), highlighting potential novel roles for endothelial Notch in G protein regulation (Fig. S2G). Notably, the Rho Family GTPase 1 *RND1* was clearly exceptional in both fold change and significance of response to Notch signaling (Fig. 1H–J).

### RND1 and rapidly-regulated genes respond to Notch in multiple signaling contexts

**Figure 2:**
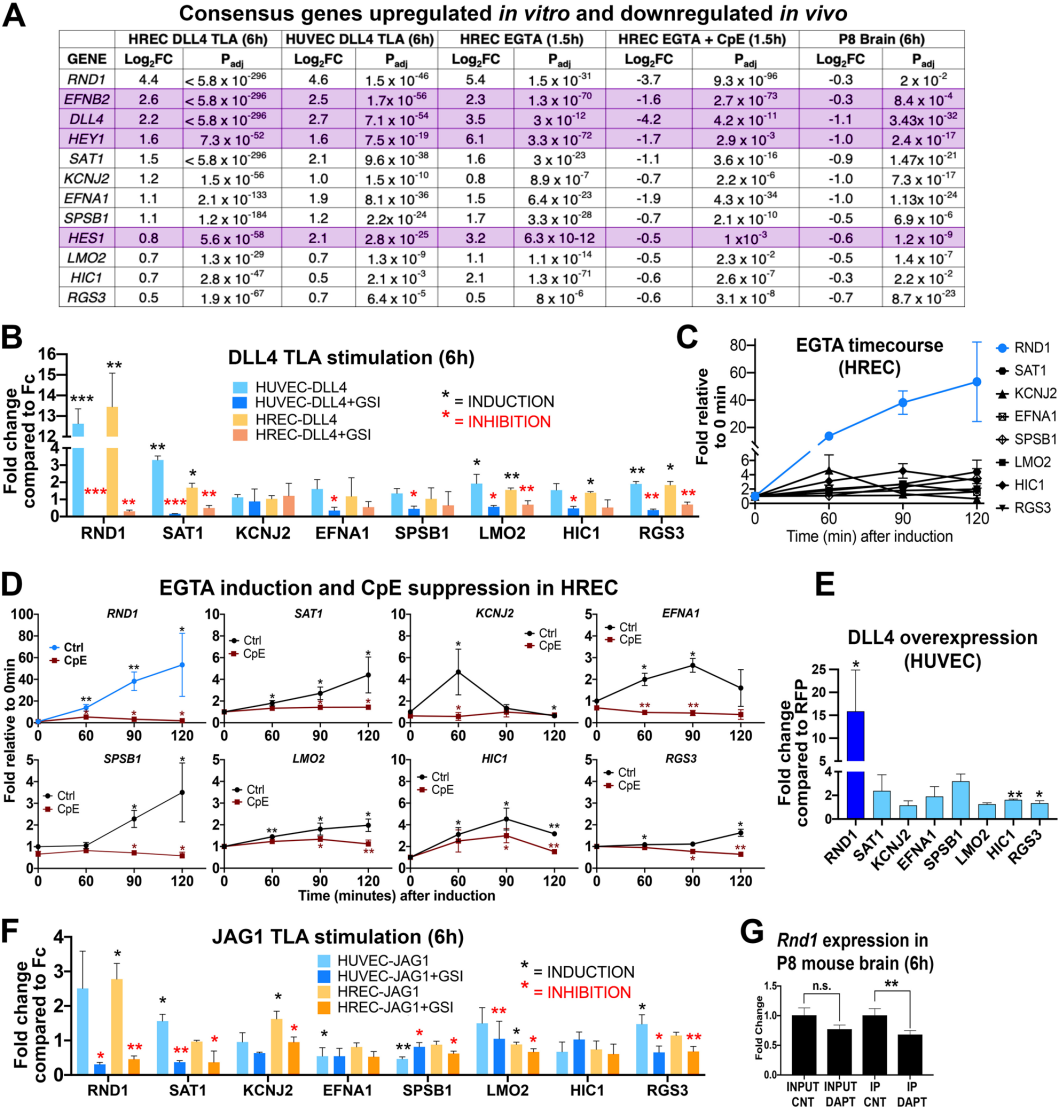
*RND1* is the most highly regulated of the novel Notch targets in multiple contexts. (**A**) 12 genes were significantly regulated under all in vitro and in vivo RNA-seq screening conditions. Genes in purple are established Notch targets. (**B**) qPCR confirms that 7 of the 8 novel genes are regulated by DLL4 TLA in HUVEC and HREC, with *RND1* showing the highest fold induction and the strongest inhibition by CpE. Black stars indicate significant induction by DLL4, red stars indicate significant inhibition of induction by CpE. (**C**) All novel targets are induced by EGTA; the most strongly induced gene is *RND1* (blue line)*.* (**D**) EGTA induction (blue or black lines) and CpE inhibition of EGTA (red lines) of the 8 novel putative endothelial Notch targets in HREC. Black stars indicate significant induction, red stars indicate significant inhibition of induction. The y axes are scaled for each gene to visualize induction of different magnitudes. (**E**) *RND1, HIC1*, and *RGS3* remain induced after 48 h of DLL4 overexpression, while the other novel targets have transient expression that has returned to baseline after 48 h. (**F**) JAG1 TLA induces expression of most novel endothelial Notch targets at lower levels, suggesting that *RND1* is a target of both DLL4 and JAG1 signaling. (**G**) qPCR of endothelial transcripts immunoprecipitated from the P8 mouse brain using an endothelial-specific RiboTag allele. Input = bulk brain homogenate, IP = immunoprecipitated endothelial mRNA.

To confirm physiologic relevance in vivo, we examined endothelial transcripts from postnatal day 8 (P8) mouse brain using a RiboTag model^27,28^*.* Changes in the overall transcriptional profile of the P8 mouse brain will be described in a separate publication. Briefly, P8 pups were treated for 6 h with GSI DAPT to inhibit Notch signaling and endothelial transcripts were isolated from whole brain homogenates by immunoprecipitating endothelial-specific HA-tagged ribosomes. DAPT shows slightly different GSI activity than CpE at low concentrations, but both compounds exhibit strong inhibition of Notch cleavage and Notch signaling at the concentrations used in these experiments^29^. Comparison of the in vitro and in vivo rapidly regulated genes gave us a consensus set of 12 rapidly regulated genes (Fig. 2A). We examined the 12 consensus genes to determine if they are significantly regulated under a variety of in vitro and in vivo conditions. Of the consensus genes, 4 are previously established Notch targets (*EFNB2*, *DLL4*, *HEY1*, *HES1*), validating that our approach identifies Notch targets. The remaining 8 consensus genes have not previously been implicated in the Notch pathway and were not observed in analyses where constitutive Notch activation was examined in endothelial cells^4,6,20,22^.

We validated the 8 novel Notch target genes for response to a variety of Notch signaling activations. For all assays that had previously been analyzed by RNA-seq, we repeated experiments with independent batches of endothelial cells for qRT-PCR. Notch-responsive expression of candidate genes in HUVEC and HREC was confirmed using a DLL4 TLA assay to activate Notch signaling and the gamma-secretase inhibitor CpE to confirm Notch specificity. Of the 8 novel genes, 7 genes showed significant induction by DLL4 and/or repression by CpE after 6 h (Fig. 2B). *RND1* had the highest magnitude of upregulation and was strongly repressed by GSI in both EC types, suggesting that *RND1* is primarily under control of Notch signaling (Fig. 2B and S3A). Each of the 8 novel genes showed significant induction within two hours after EGTA addition when assessed by qPCR, but the magnitude and kinetics of induction varied considerably (Fig. 2C,D and Fig. S3A). *KCNJ2*, *EFNA1*, and *HIC1* showed a rapid peak and then return towards baseline within 2 h, which may explain their relatively modest induction when examined after 6 h in the TLA assay. The other genes showed a steady increase in induction after EGTA addition, with *RND1* again showing high magnitude induction. EGTA induction was significantly inhibited by CpE for all genes (Fig. 2D). Similar results were observed in HUVEC (Fig. S3A).

To determine whether the 8 novel endothelial Notch targets are regulated by constitutive Notch signaling, we examined expression levels in HUVEC constitutively overexpressing DLL4 for 48 h. *SAT1*, *KCNJ2*, *EFNA1*, *SPSB1*, and *LMO2* were no longer significantly upregulated compared to RFP-infected controls, demonstrating that rapid response screening identifies unique Notch targets that may not be maintained under steady-state Notch signaling conditions (Fig. 2E). Induction of *RND1* was maintained at approximately 15-fold above control levels under constitutive Notch signaling conditions, similar to the approximately 12-fold induction observed after 6 h in the TLA assay, suggesting that it is a target of both rapid and constitutive Notch signaling.

While DLL4 strongly activates endothelial Notch signaling and suppresses EC sprouting, the Notch ligand JAG1 is pro-angiogenic and has been variously proposed to function as weak endothelial Notch activator or a suppressor of DLL4/Notch signaling^30–32^. When we conducted a TLA using 40ug/ml JAG1-Fc, JAG1 significantly induced almost all of the novel Notch targets, including *RND1*, in at least one endothelial cell type, although the magnitude of induction was generally low (Fig. 2F). *RND1* was activated 2–threefold by JAG1 signaling, in comparison to 12–14 fold activation by DLL4 signaling. These data suggest that JAG1 indeed activates endothelial Notch signaling and regulates many Notch targets that are regulated by DLL4, albeit at lower magnitude effect.

To confirm the in vivo relevance of *RND1*, we examined translated RNA from P8 endothelial RiboTag mouse brains treated with DAPT. Bulk brain homogenate (Input) showed no significant difference, suggesting that RND1 expression is Notch-independent in neuronal tissues, but endothelial cells showed a significant drop in *RND1* mRNA, confirming that *RND1* is rapidly regulated by Notch in the brain endothelium in vivo (Fig. 2G).

### RND1 is a direct Notch target that facilitates Notch-mediated suppression of endothelial Ras activity

**Figure 3:**
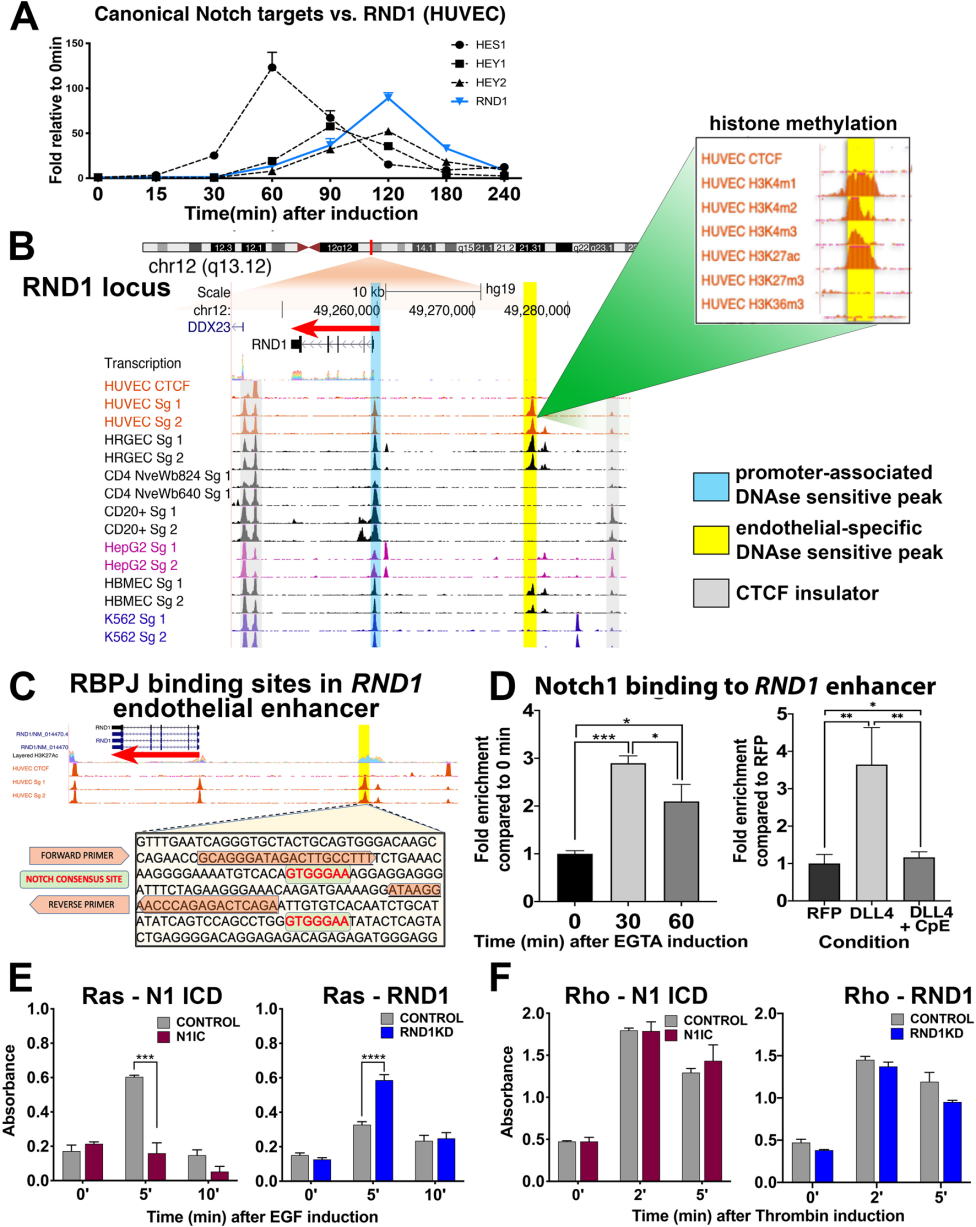
The RND1 locus contains an active endothelial specific enhancer with Notch binding sites. (**A**) *RND1* is induced by EGTA at similar rates and magnitude as well-characterized *HES* and *HEY* Notch targets. (**B**) ENCODE database view of the *RND1* locus shows a DNAse hypersensitivity peak at the promoter region in all cell types (blue bar) and a second peak upstream that appears only in endothelial cell types (yellow bar). The endothelial-specific peak shows histone methylation patterns consistent with active enhancers (insert box). Gray bars mark CTCF-binding insulator regions. (**C**) Enhancer region sequence in the RND1 locus with two Notch consensus sequence (green highlight). Also highlighted is the primer sequence for ChIP PCR (orange highlight). (**D**) ChIP assay of the putative endothelial-specific *RND1* enhancer with antibodies against the intracellular domain of Notch1 (N1ICD). N1ICD binds to the *RND1* enhancer after EGTA induction (left) or DLL4 overexpression, but binding is blocked with CpE treatment, indicating that active Notch signaling is required. (**E**) HUVECs transfected with siCon or siRND1 or infected with Control or Notch1IC lentiviral constructs were serum-starved for 3 h, treated with 100 ng/ml EGF, and analyzed for Ras activity by G-LISA after 0, 5, and 10 min. (**F**) HUVECs as in (E) were treated with 50 nM thrombin and analyzed for RhoA activity by G-LISA after 0, 2, and 5 min.

The various manipulations of Notch signaling that we tested dramatically induced or repressed *RND1*. We therefore determined whether *RND1* is a direct or an indirect target of Notch signaling. EGTA activation of Notch signaling induced *RND1* in a similar timeframe as known direct Notch targets (Fig. 3A). Examination of the ENCODE database indicated that open chromatin DNAse hypersensitivity (DHS) peaks were present near the RND1 transcriptional start site in a broad array of cell types, consistent with a promoter that facilitates expression of *RND1* in diverse cell types [Fig. 3B, https://genome.ucsc.edu/encode/;^33^]. A separate DHS peak ~ 17 kb upstream from *RND1* was observed only in endothelial cell types, suggesting that this region encodes an endothelial-specific *RND1* enhancer (Fig. 3B). The putative EC enhancer exhibited histone marks typical of active enhancers^34^ and contains two RBPJ/CSL-binding consensus sequences (C/T)GTGGGAA, suggesting that this region may mediate Notch-responsive transcriptional regulation in EC (Fig. 3B,C)^35,36^.

To determine whether the putative EC *RND1* enhancer is bound by Notch, we conducted chromosome immunoprecipitation (ChIP) assays with antibodies against the Notch1 intracellular domain followed by PCR to detect the EC enhancer. In HUVEC, Notch1ICD bound to the EC *RND1* enhancer within 30 min after EGTA treatment (Fig. 3D). Notch1ICD also bound to the EC *RND1* enhancer in response to DLL4 expression in a GSI-repressible manner, demonstrating that binding depends on Notch signaling (Fig. 3D). We conclude that *RND1* is a direct Notch target and regulation of *RND1* may be facilitated by an endothelial enhancer found upstream of the transcriptional start site.

Examination of the remaining novel consensus genes in the ENCODE database identified putative endothelial-specific enhancers containing consensus or alternate RBPJ binding motifs in *SAT1*, *KCNJ2*, *EFNA1*, *SPSB1*, and *RGS3* (Fig. S1B-C)^21,37^. Thus, we identified several other Notch target genes that rapidly respond to diverse means of Notch activation and whose regulatory elements include likely endothelial Notch-responsive enhancer elements, although their roles in angiogenesis are not clear in all cases. Several of the genes identified, such as KCNJ2 and LMO2 respond to VEGF-A^38–41^, suggesting that VEGF-induced activation of Notch signaling is critical for their regulation. *SAT1* is enriched in tumor endothelial stalk cells, where Notch signaling is highly active^42^, and encodes a key regulator of polyamine content^43^, suggesting it may participate in Notch control of endothelial metabolism.

RND1 is a member of the Rho GTPase family which regulates the organization of actin cytoskeleton, cell migration, and cell adhesion^44–47^. Current studies of RND1 activity in HUVEC are conflicting: it has been reported to inhibit Ras activation with no effect on Rho activity, or to inhibit RhoA activation^48,49^. We observed that Notch activation in HUVEC significantly reduced Ras activation in response to EGF^50^, whereas RND1 knockdown significantly promoted Ras activation (Fig. 3E). We conclude that Notch suppression of Ras activity is facilitated, in part, by RND1. By contrast, Rho activation by thrombin^51^ was unaltered in HUVEC by Notch activation or siRND1, suggesting that Notch signaling does not affect this pathway (Fig. 3F).

### Notch regulation of endothelial migration is mediated by RND1

**Figure 4:**
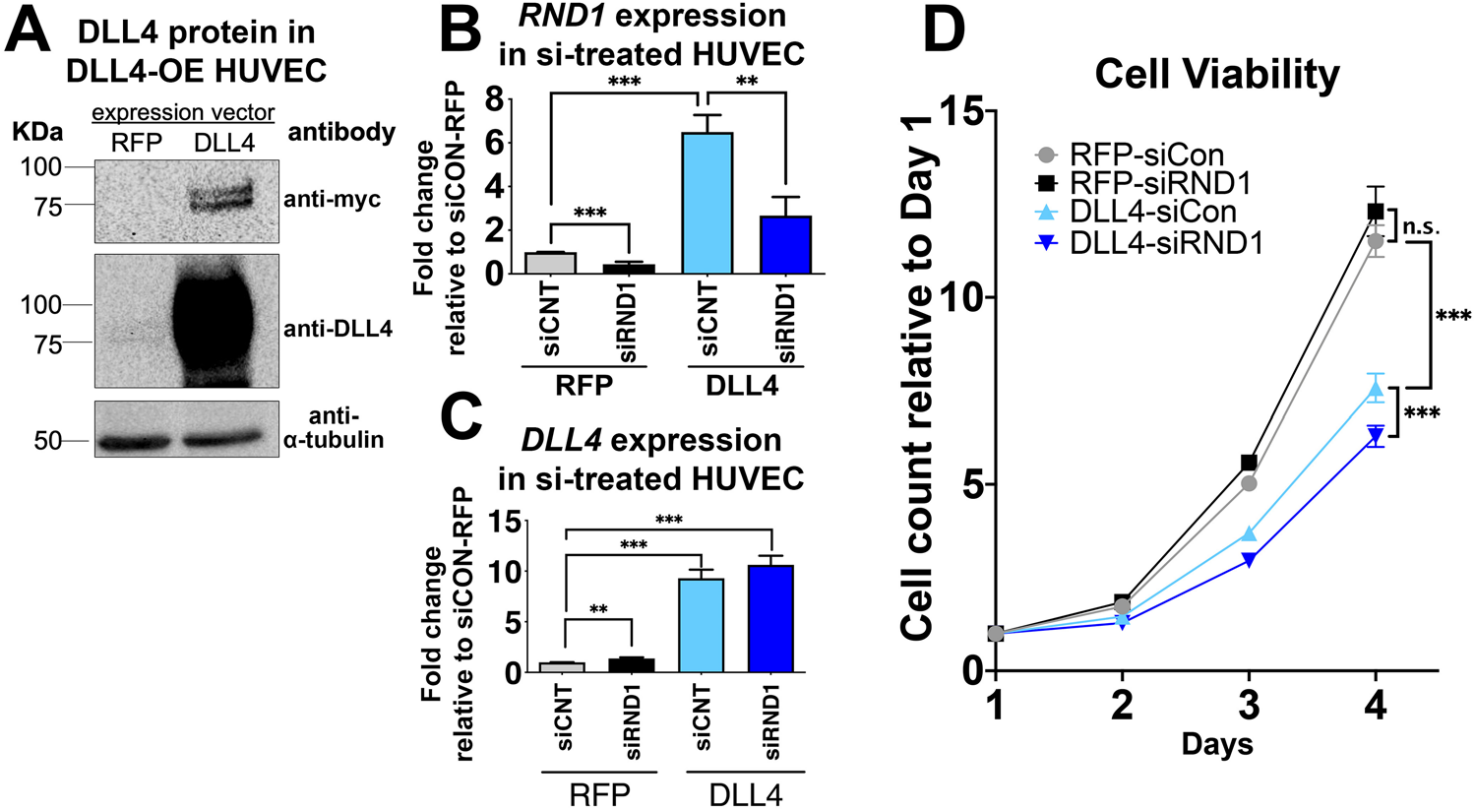
RND1 knockdown does not rescue Notch-mediated suppression of endothelial proliferation. (**A**) Transfection with a DLL4-myc expression construct induces DLL4 protein expression in HUVECs 3 days after lentivirus induction. All images are from the same blot, with uncropped version provided in Fig. S4. (**B**) RND1 siRNA robustly knocks down RND1 in HUVEC with normal levels of Notch signaling (RFP) or Notch signaling activated by DLL4-myc overexpression (DLL4). HUVECs were lentivirally transduced with RFP or DLL4 and transfected the next day with scrambled (siCNT) or siRNA targeting RND1 (siRND1). 3 days after lentivirus infection, HUVECs were harvested and analyzed by qPCR for RND1. (**C**) Treatment with siRNA1 does not grossly affect DLL4 levels in HUVEC treated as in (**B**). (**D**) Treatment with siRND1 does not restore proliferation in HUVEC where Notch signaling has been activated by DLL4. 10^4^ HUVEC treated as in (**B**) were seeded onto 6-well plates cell and counted daily. Proliferation rate was normalized to counts at day 1.

To determine if RND1 mediates Notch regulation of angiogenesis, we overexpressed DLL4 (DLL4 OE, Fig. 4A) to activate Notch and induce angiogenic responses and knocked down RND1 to establish whether this reduced the magnitude of Notch response. siRNA targeted against *RND1* (siRND1) reduced *RND1* transcript levels by approximately 70% at 24 and 48 h post-transfection, indicating a partial loss of RND1 function when angiogenic assays were initiated (Fig. 4B,C). DLL4 expression suppressed endothelial cell proliferation, indicative of Notch activation (Fig. 4D). RND1 knockdown did not substantively alter the growth rate of HUVEC or reverse the effects of DLL4 (Fig. 4D). We conclude that RND1 does not mediate endothelial growth suppression by Notch signaling.

**Figure 5:**
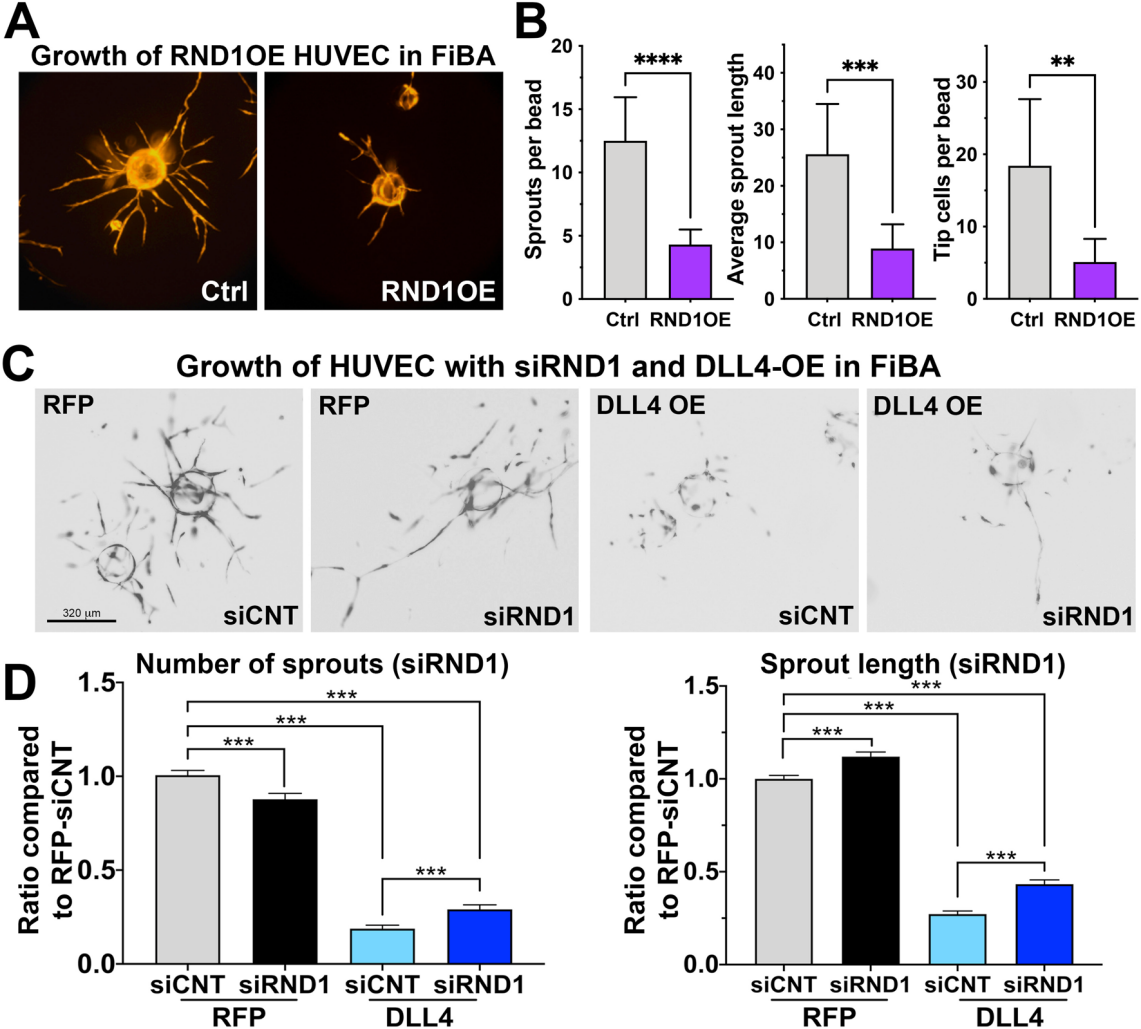
RND1 is required for Notch-mediated suppression of sprout number and length. (**A**) Representative images of Fibrin bead angiogenesis (FiBA) assays using lentivirally-transduced HUVECs with full-length *RND1* overexpression vectors (RND1OE) or empty vector (Ctrl). HUVEC were coated on cytodex beads, embedded in a fibrin gel with fibroblasts (D551) grown on the surface of the gel to provide growth factors, and allowed to develop for 5 days. (**B**) After 5 days, overexpression of *RND1* reduces the number of sprouts, length of sprouts, and number of tip cells in a highly significant manner. (**C**) Lentivirally-transduced HUVECs with RFP or DLL4 expression constructs and were transfected with siCNT or siRND1 used for FiBA assays as above. Representative images of FiBA under each condition, scale bar indicates 320 µm. (**D**) After 5 days, Notch activation by DLL4 suppresses the number of sprouts from each bead, but knockdown of RND1 partially rescues sprout number (left). Notch activation by DLL4 suppresses the length of sprouts from each bead, but knockdown of RND1 partially rescues sprout length (right).

Notch signaling normally suppresses endothelial sprouting. We tested the effect of overexpression of *RND1* in a 3D angiogenesis assay where endothelial cells are coated onto beads, which are then embedded in to fibrin gel (Fibrin Bead Assay or FiBA)^52^. Overexpression of *RND1* dramatically reduced the number and length of endothelial sprouts and the number of sprouting tip cells (Fig. 5A,B). We therefore tested whether loss of RND1 can overcome Notch inhibition of sprouting. Notch signaling activation via DLL4 overexpression resulted in significant reduction of sprout numbers and length in FiBA assays, as expected (Fig. 5C,D). siRND1 resulted in a modest but significant restoration of sprout numbers and increased sprout length in Notch activated endothelial cells, demonstrating that sprout extension is suppressed in part by RND1 (Fig. 5C,D).

**Figure 6:**
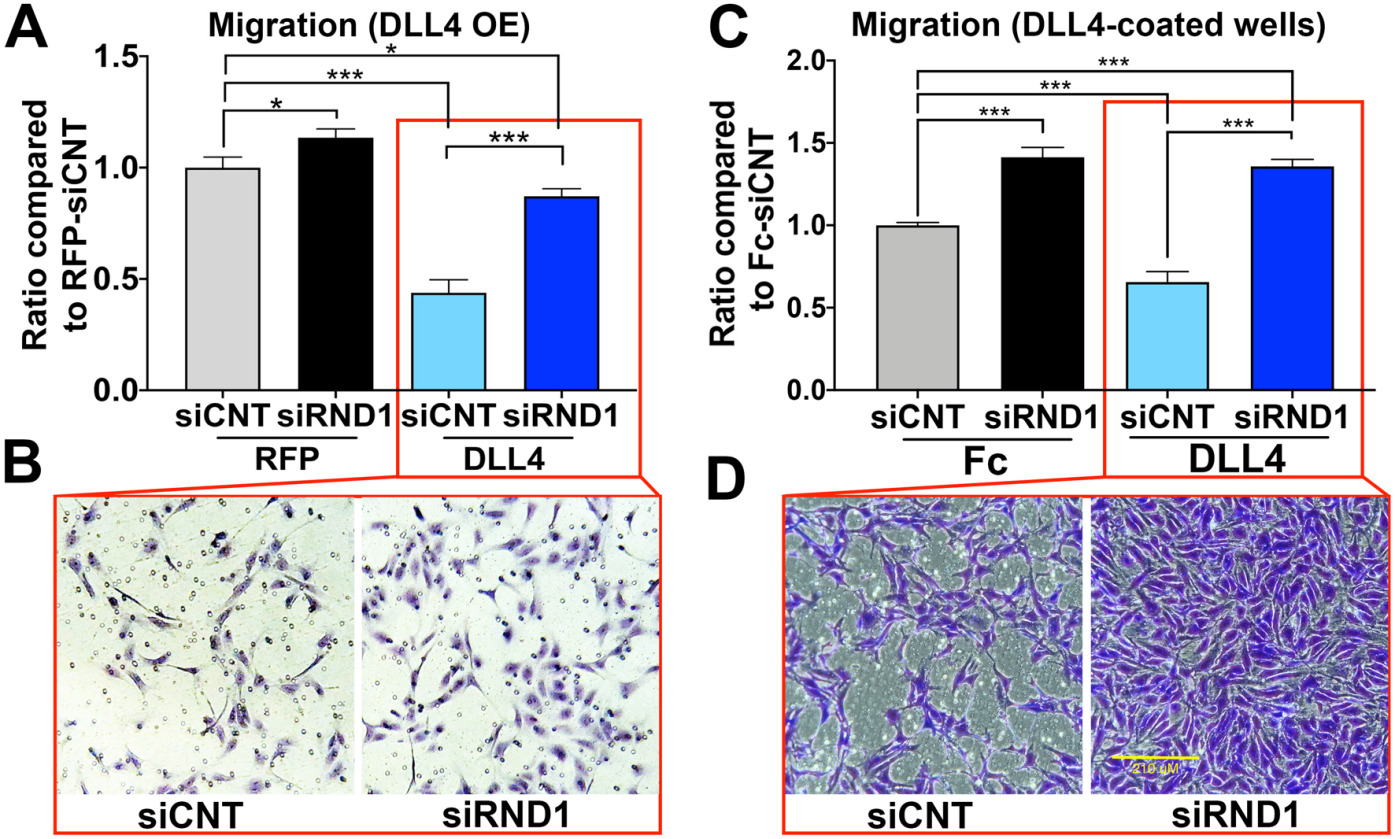
RND1 is required for Notch-mediated suppression of EC migration. (**A**) Lentivirally-transduced HUVECs with RFP or DLL4 expression constructs were transfected with siCNT or siRND1 and plated in modified Boyden chambers and incubated with 50 ng/ml of VEGF for 6 h. Cells that had migrated through the pores were counted and normalized to their respective controls. DLL4 overexpression activation of Notch signaling suppresses endothelial migration (third column), but knockdown of RND1 rescues the endothelial migration to nearly control levels. (**B**) Representative images of cells that had migrated through Boyden chamber pores under each condition in (**A**). (**C**) Control and siRND1 transfected HUVECs were plated on a Boyden chamber inserts coating with fibronectin (5 µg/ml) and either Fc (control) or 10 µg/ml DLL4-Fc (Notch activation) and incubated with 50 ng/ml of VEGF for 6 h. Physiologic activation of Notch signaling by tethered DLL4 ligand suppresses endothelial migration (third column), but knockdown of RND1 increases migration under endogenous Notch signaling conditions (second column) or ligand-induced Notch signaling (fourth column). (**D**) Representative images of cells that had migrated through Boyden chamber pores under each condition in (B). The scale bar indicates 210 μm.

Notch activity suppresses endothelial migration and limits sprout extension via migration^53^. When Notch was activated via overexpression of *DLL4*, siRND1 knockdown substantially restored the ability of endothelial cells to migrate through transwell filters in response to VEGF (Fig. 6A,B). To ensure that *DLL4* overexpression did not introduce artifacts, we repeated these experiments using DLL4-coated filters to activate Notch, which models physiologic paracrine presentation of DLL4, and confirmed that siRND1 restored the level of migration to control levels (Fig. 6C,D). These results indicate that Notch signaling requires RND1 as an effector protein to suppress VEGF-induced migration and sprout extension. We conclude that *RND1* is a novel Notch effector necessary for Notch suppression of EC migration and links Notch to the Ras G-protein signaling pathway.

## Discussion

**E**ndothelial Notch signaling has distinct roles in stalk cells that must be controlled temporally; initially, Notch suppresses the tip cell phenotype, thus stimulating stalk cell attributes, this then allows Notch to subsequently promote vessel maturation. Tip and stalk cells have the ability to change places dynamically, depending on the level of Notch signaling^54,55^ implying that suppressing the tip cell phenotype may be coordinated with control of migration. In this study, we have identified a cohort of genes regulated by endothelial Notch within hours of signal activation or inhibition, revealing a previously underappreciated set of rapidly induced genes that are candidate effectors controlling dynamic tip/stalk regulation.

The endothelial Notch transcriptomes we identified include several GPCR regulatory proteins and GTPases, which directly control a multitude of cellular phenotypes and are excellent candidates for rapid phenotypic change but have not previously been implicated as Notch effectors. However, an overlapping but distinct set of GPCR proteins were regulated in each cell type examined, suggesting that endothelial cells from different vascular beds may employ unique GPCR effectors.

We posited that Notch signaling regulates separate effectors to control different endothelial cell phenotypes. Among the putative effector GTPases discovered, the small GTPase *RND1* was consistently upregulated by Notch signaling in all cell types and signaling conditions examined, and was generally one of the most strongly regulated genes. We established that RND1 is a direct Notch target and that RND1 is required for Notch-mediated suppression of sprout extension, migration, and Ras activity, but not proliferation, confirming its role as an effector of specific Notch phenotypes.

To capture how Notch signaling carries out its diverse functions in endothelium, further examination of the rich set of novel, candidate Notch effectors identified by this study will facilitate greater understanding of Notch function in angiogenesis. RND1 was demonstrated to facilitate the inhibition of migration by Notch, but other candidates likely facilitate other cellular responses, such as proliferation, filopodia suppression, and promotion of barrier function. An appreciation of the “first wave” of rapidly regulated effectors that drive the role of Notch in sprouting angiogenesis will undoubtedly guide future design of therapeutic intervention in the Notch pathway.

## Methods

### Endothelial cell and tissue culture

Primary Human Umbilical Vein Endothelial Cells (HUVECs) were isolated by the Kitajewski lab from de-identified discarded human umbilical cords, which has been determined by the UIC Office for the Protection of Research Subjects (OPRS) to not meet the definition of humans subjects research. All OPRS guidelines were followed. HUVECs were isolated following established protocols^56^ and cultured in EGM-2 without hydrocortisone (Lonza, Cat#CC-3162) on dishes coated with rat tail type I collagen (Corning, Cat#354249). Endothelial identity was confirmed by assessing cobblestone morphology and VE-cadherin/CD31 double positivity at time of isolation (anti-CD31: Invitrogen MA5-13188, 1:100, anti-VE-cadherin: Cayman 160840, 1:100). To ensure retention of endothelial properties during culture, all HUVEC were used at passage 5 or lower, and cobblestone morphology and VE-cadherin/CD31 staining was re-assessed at passage 6 (Fig. S1). HUVEC batches A, A1, and B were used for experiments in Figs. 1, 2 and 3, batches U1 and U2 were used for experiments in Fig. 4.

Primary Human Retinal Microvascular Endothelial Cells (HREC, Cat. ACBRI 181, Passage 3) were obtained from a single vial purchased from Cell Systems, which validated these HREC for expression of CD31, VWF, and uptake of Ac-LDL (greater than 99% purity). HREC were cultured in EGM-2 with all bullet kit components on dishes coated with 0.2% gelatin (Sigma, G1393) at passage eight or lower. Retention of endothelial identity was re-assessed as above at passage 14 (Fig. S1). HEK293T cells and Detroit 551 (D551) human skin fibroblasts were purchased from ATCC and maintained DMEM (Gibco, Cat No. 11-995-073) with 10% FBS.

### Tethered ligand assay (TLA)

The recombinant extracellular domains of the Notch ligands hDLL4-Fc (Cat. 10171-H02H, Sino Biologicals Inc.), hJAG1-Fc (Cat. 11648-H02H, Sino Biologicals Inc.) or IgG-Fc (Cat. 10702-HNAH, Sino Biologicals Inc.) were coated on 24-well plates (Corning, P/N 353226) in Fibronectin matrix (Cat. F1141, Sigma). Following an overnight incubation at 4 °C, primary ECs at 80% confluency were trypsinized and seeded onto the coated plates and incubated at 37 °C with 5% CO_2_ for indicated amount of time. Candidate targets identified by RNAseq were validated by RT-qPCR on a different frozen batch of HUVECs or HRECs.

### RNA isolation and RT-qPCR

RNA from the cells or tissues were extracted using the RNeasy Mini Kit (Qiagen, Cat. 74104) as per the manufacturer’s recommendations. Reverse transcription was done using the Verso cDNA synthesis kit (Fischer Scientific, Cat. AB-1453/B) and target gene expression was assessed by Quantitative Real Time PCR (qPCR) using SYBR Green master mix (Applied Biosystems, Cat.4385612) and primers specific to genes of interest (Supplemental Table 1).

### EDTA/EGTA Notch activation assay

Primary ECs at 70% confluency were treated with compound E (CpE, Cat. ALX-270–415-c250, Enzo Life Sciences) at 200 nM overnight to inhibit endogenous Notch signaling. The following day, cells were washed with PBS 2X and treated with 1XPBS with 10 mM EGTA for 15 min at 37 °C. After incubation, the PBS was replaced with fresh EGM-2. Start time was established as the moment that EGTA was added. RNA was collected as outlined above at time points from 30 min up to 4 h. For the CpE treatment group, 500 nM of CpE was added along with the EGTA at 0 h.

### P8 RiboTag-based isolation of brain endothelial mRNA

All animal experiments were approved in advance by the University of Illinois Animal Care Committee and comply with the USPHS Policy on Humane Care and Use of Laboratory Animals and we report these methods in compliance with the ARRIVE guidelines (https://arriveguidelines.org/arrive-guidelines/experimental-animals). All mice were maintained on a congenic C57BL6/J background. Homozygous *Rpl22*^*tm1.1Psam*^; *Cdh5-CreERT2* mice were mated and the day pups were first observed was assigned as postnatal day 0 (P0). Nursing females were gavaged with 250 mg/kg tamoxifen in oil at postnatal day (P) P1, P2 and P3 to induce Cre recombination. Recombination efficiency and tissue specificity of Rpl22HA expression was confirmed by sectioning the brains of P5 pups and immunostaining for HA. P8 pups (approximately 3.5 g each) of both sexes were randomized and injected subcutaneously with a single dose of 100 mg/kg DAPT (20 mg/ml in 10% ethanol 90% corn oil) or vehicle alone. Pups were humanely euthanized 6 h after injection via decapitation with a sharp blade and brains were snap frozen and homogenized. Endothelial-specific transcripts were obtained by immunoprecipitating RiboTag labeled ribosomes with anti-HA antibody (1:200, ab9118, Abcam) and purifying the resulting RNA using a Qiagen RNeasy mini kit. All genotyping was performed by Transnetyx.

### Generation and validation of DLL4 and RND1 overexpressing HUVECs

To activate Notch signaling, we created a human DLL4 overexpression lentivirus vector (pCCL-PGK-DLL4) consisting of human DLL4 with C-terminal myc-6xHis-Tag in a pCCL vector and used tdTomato (RFP) expressing vector as a control. Lentivirus was generated in HEK293T cells through co-transfection of lentiviral packaging (pMDLg/pRRE and pRSV-Rev) and envelop vector (pCMV-VSV-g) with pCCL-PGK-RFP or pCCL-PGK-DLL4. For transduction with lentivirus, HUVECs were incubated with EGM-2 and the supernatant of the lentivirus infected HEK293T cells (2:1 ratio mixture) overnight and changed to fresh EGM-2 media the next day. Infection rate was estimated using RFP expression in cells. After 2 days, HUVECs were harvested with RIPA buffer (50 mM Tris (pH8.0), 150 mM NaCl, 2 mM EDTA, 1% Triton X-100, 0.5% Sodium deoxycholate, 0.1% SDS) with Halt protease inhibitor cocktail (Invitrogen, Cat no. 78430). 20–40 μg of protein was loaded on 7% SDS-PAGE and DLL4 expression was evaluated with anti-myc antibody (1:1000 dilution, Sigma, Cat no. 06-549), anti-DLL4 antibody (1:1000 dilution, Cell Signaling, Cat no. 2589) and anti-α-tubulin (1:2000 dilution, Sigma, Cat No. T6074). Anti-rabbit-HRP (1:3000 dilution, Cell Signaling, Cat no. 5127) for myc and DLL4 and anti-mouse-HRP (1:3000 dilution, GE Amersham. Cat No. NA9310) were used and the specific signals were developed with ECL (GE Amersham. Cat No. RPN2209) and detected by ChemiDoc MP (Bio-Rad). To overexpress *RND1*, lentivirus containing pCCL-RND1, consisting of full-length unaltered human *RND1*, or empty pCCL vector were packaged and used to infect HUVEC as above.

### Chromatin immunoprecipitation (ChIP) assay

3 × 10^6^ HUVECs were treated with 10 mM EGTA for 15 min, then changed to new EGM-2 for 0, 30, and 60 min prior to fixation. Alternately, HUVECs were transduced with lentivirus-RFP or DLL4 and 3 × 10^6^ cells were treated with 500 nM γ-secretase inhibitor (CpE) or vehicle control, incubated overnight, and fixed. Cells were fixed with 1% PFA at room temperature for 10 min. Cell nuclei were extracted using NP40 buffer (10 mM Tris (pH8.0), 10 mM NaCl, 0.5% NP40), lysed with SDS buffer (50 mM Tris (pH8.0), 1% SDS, 10 mM EDTA), and sonicated to an approximate DNA fragment length of 100–500 bp. Activated Notch intracellular domain (N1ICD) was precipitated by incubating the sheared genomic DNA with anti-cleaved Notch1 antibody (1:200 dilution, Cell signaling, Cat no. 4147) and Dynabeads Protein G (Invitrogen, 10003D) for 2 h at 4 °C. DNA was purified (ThermoFisher, Cat no. K0832) and probed for the *RND1* enhancer with quantitative realtime PCR (qPCR). The N1ICD binding capacity was quantified by calculating the relative abundance between the experiment and control samples after normalizing to the respective inputs using the 2^ΔΔCt^ method.

### Fibrin bead assay (FiBA)

HUVECs were infected with empty vector, RND1OE, RFP or DLL4OE lentiviruses, then transfected with siRNA (control or *RND1*, 25 nM) using DharmaFECT 4 (Dharmacon, Cat no. T-2004-03) and trypsinized the following day. For FiBA, 6 × 10^4^ cells were incubated (5% CO_2_, 37 °C) for 4 h with approximately 150 cytodex3 beads (5ul of 30 beads/ul suspension; Sigma, Cat No. C3275) suspended in EGM-2 to generate HUVEC-coated beads. HUVEC-coated beads were cultured on a 6-well plate overnight to eliminate loose HUVECs. HUVEC-coated beads were embedded in fibrin gel made with fibrinogen (3 mg/ml, Sigma, Cat No. F8630), aprotinin (1.5 TIU, Sigma, Cat No. A3428) and thrombin (312.5 mU, Sigma, Cat No. T4648). After gel formation, human dermal fibroblasts (D551, 1 × 10^5^ cells) were seeded on top of the fibrin gel and supplemented with fresh EGM-2 media every day for 5 days. On the 5th day, the RFP positive sprouts from coated beads were assessed for the length and numbers of sprouts from 30 beads/group in three independent experiments using Image J.

### VEGF induced migration

To evaluate Notch suppression of cell migration we overexpressed DLL4 or stimulated HUVECs with tethered DLL4-Fc recombinant protein. In the first approach, HUVECs transduced with lentivirus encoding RFP or DLL4 were administrated siRNA (control or *RND1* siRNA, 25 nM) to knockdown *RND1* expression, as described above. HUVECs were cultured overnight in the absence of added serum and VEGF in EGM-2 containing 0.2% FBS but no VEGF. 6–8 × 10^4^ HUVECs were seeded on the upper chamber of a 0.1% gelatin coated 8 μm pore cell culture inserts (Fisher, Cat no. 08-771-21). In the second approach, 6–8 × 10^4^ HUVECs transfected with control or *RND1* siRNA as above were seeded on the upper chamber of 8 μm pore cell culture inserts coated with fibronectin (10 μg/ml, Cat. F1141, Sigma) mixed with human Fc or DLL4-Fc (10 μg/ml, Sino Biologicals Inc.) as described in the TLA assay.

In both assays, additional VEGF (50 ng/ml, R&D Cat no. 2179-VC-025/CF) was added to EGM-2 (containing 0.2% FBS but no VEGF) in bottom chamber. Cells were incubated for 6 h, fixed with 4% PFA (EMS, Cat no. 15712) for 10 min, and stained with 1% Crystal violet (Sigma, Cat no. C0775). Cells that had migrated through the pores and were adherent to the lower side of the insert were imaged to 5–7 different 10X fields per group. Three independent experiments were counted with imageJ and normalized to the corresponding control.

### Detection of Ras activation and Rho activation

HUVECs were grown overnight in EBM-2 (containing 1% FBS) and changed to serum-free EBM-2 for 3 h the following day. The cells were then stimulated with 100 ng/ml hEGF for 5–10 min (Sigma-Aldrich, Cat No. E9644) for Ras activation, or with 50 nM Thrombin for 2–5 min (Enzyme Research Laboratories, Cat No. HT1002a) for RhoA activation. Assays were performed using RhoA (Cytoskeleton, Cat No. BK124), and Ras (Cytoskeleton, Cat No. BK131) G-Lisa Activation Assay Kits according to manufacturer’s recommendations and signal was measured at 490 nm absorbance.

### Proliferation

HUVECs were transduced with lentivirus-RFP or DLL4 and transfected with control or *RND1* siRNA (25 nM) as described above. 1 × 10^4^ HUVECs were seeded on collagen coated 6 well plates and grown for indicated time. 30 fields per plate were imaged with 4X lens in three independent experiments. The cell growth rate was normalized to day 1 cell numbers.

### RNA sequencing and data analysis

RNA quantity and integrity were measured using a Bio-analyzer (Agilent TapeStation 4200, UIC Genome Research core) prior to RNA sequencing. TLA HUVEC samples were sequenced at a ~ 30 million single-end (SE) read depth with 100-base fragments on the TruSeq platform in the Sulzberger Columbia Genome Center. TLA HREC samples and EGTA assay samples were sequenced at ~ 30 million paired-end (PE) reads with 150 base-pair fragments by Novogene (https://en.novogene.com/). Raw reads from in-vitro screens (HUVEC and HRECs) were mapped to the Human database (ENSEMBL/GRCh38) using STAR (version 2.5.0a) and processed with Samtools (version 1.4.1). The counts obtained by FeatureCounts (version 1.5.2) were analyzed by DESeq2 (version 1.18.1) to identify differentially expressed genes. The RNAseq datasets generated during this study are available in the NCBI Gene Expression Omnibus repository at https://www.ncbi.nlm.nih.gov/geo (Accession number GSE163568).

### Statistics

For qPCR analysis, the ΔΔCt method^57^ was used to calculate the relative expression using following steps: (1) Normalization to reference gene: ΔCt_GOI_ = Ct_GOI_ – Ct_BA_. (2) Relative expression between conditions: ΔΔCt_GOI_ = ΔCt_EXP_ − ΔCt_CNT_. For other experiments, unless otherwise noted, t-tests or ANOVA with Bonferroni post-hoc analysis was performed on all quantified data to determine significant differences between groups using GraphPad Prism. *P* values less than 0.05 were considered statistically significant. *p*-values < 0.05 are flagged with one star (*), *p* values < 0.01 with two stars (**), and *p*-values < 0.001 with three stars (***). Unless otherwise noted, experiments were repeated at least three times.

### Ethics declaration

All animal studies were done in accordance with UIC’s Institutional Animal Care and Use Committee guidelines.

## Supplementary Information

Supplementary Information.

## Data Availability

The RNAseq datasets were processed using publicly available softwares. Raw sequencing files and the processed files are deposited in NCBI Gene Expression Omnibus repository at https://www.ncbi.nlm.nih.gov/geo (Accession number GSE163568).
