# Supplementary material for: Endothelial Notch signaling directly regulates the small GTPase RND1 to facilitate Notch suppression of endothelial migration
Source: Sci Rep. 2022 Jan 31;12:1655. doi: 10.1038/s41598-022-05666-1 (PMC8804000; doi:10.1038/s41598-022-05666-1)
Supplement: Supplementary file 1 — Supplementary Information. [file 41598_2022_5666_MOESM1_ESM.pdf]

Figure S1

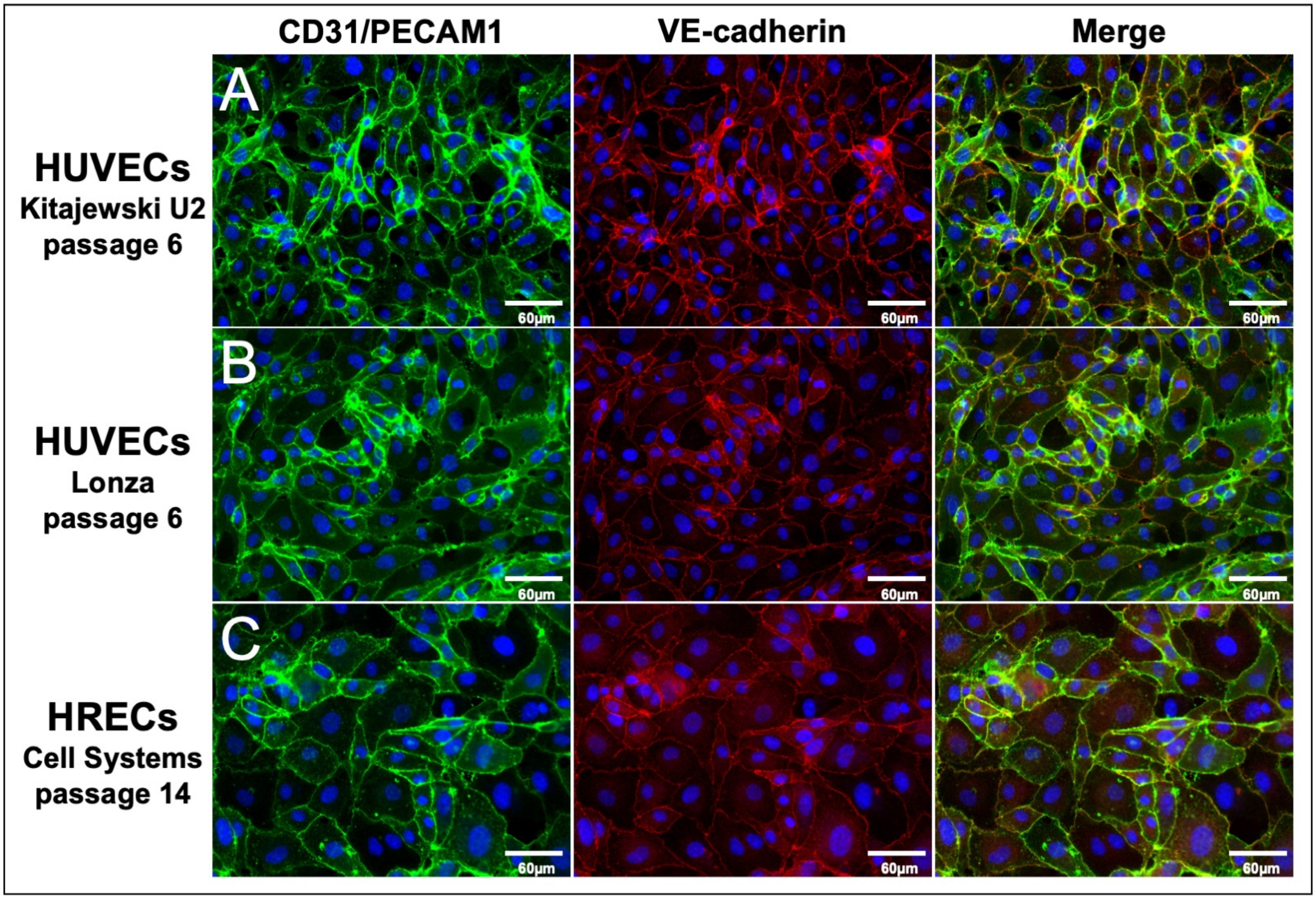

CD31/PECAM1
VE-cadherin
Merge
A
B
C
HUVECs
Kitajewski U2
passage 6
HUVECs
Lonza
passage 6
HRECs
Cell Systems
passage 14
60μm

Figure S2

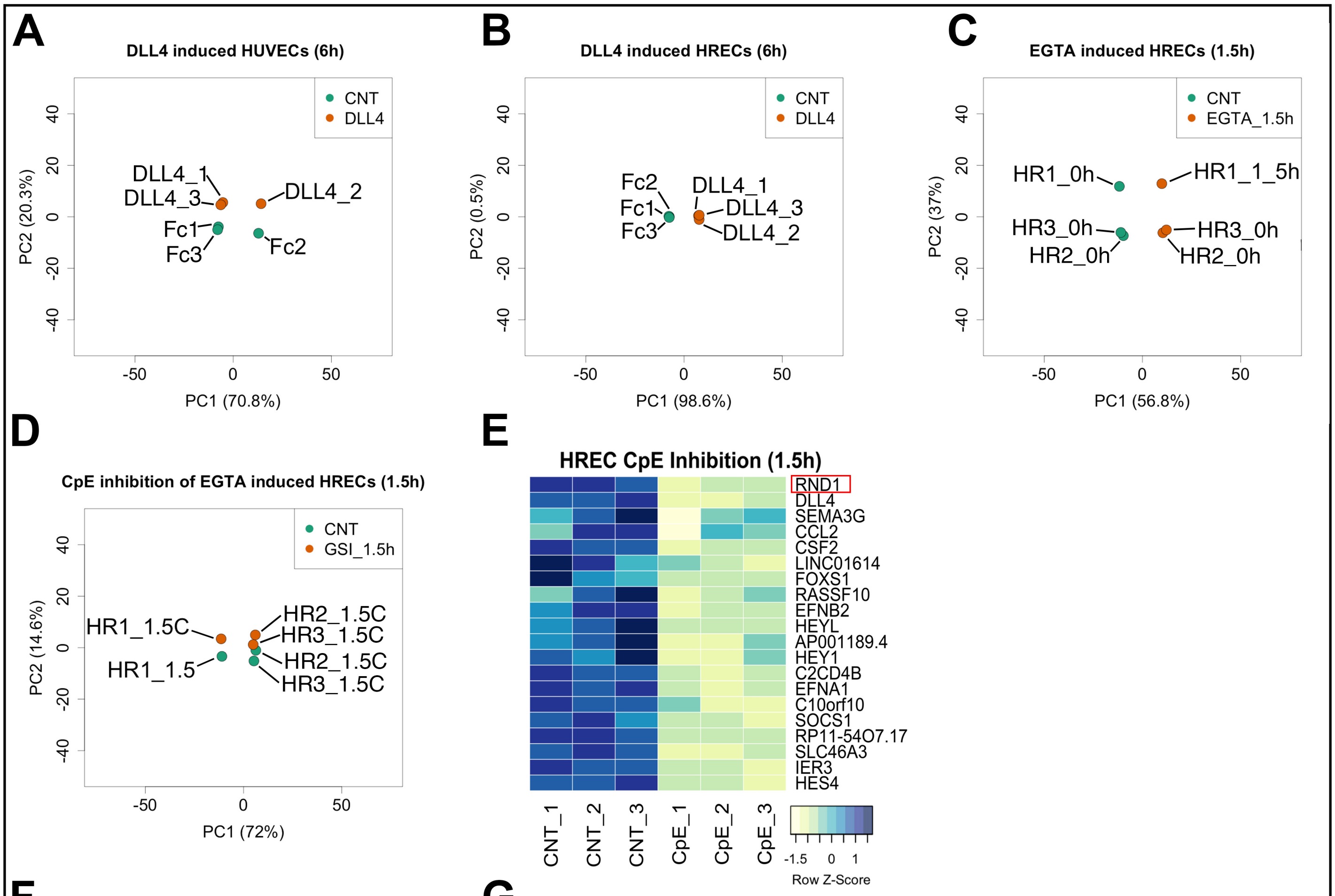

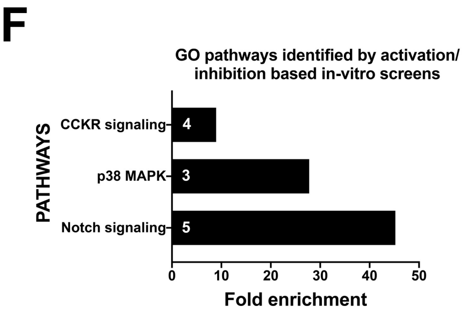

G

GENE SYMBOL	HUVEC TLA (6h) Log2FC	HUVEC TLA (6h) P_{ADJ}	HREC TLA (6h) Log2FC	HREC TLA (6h) P_{ADJ}	HREC EGTA (1.5h) Log2FC	HREC EGTA (1.5h) P_{ADJ}	HREC CpE (1.5h) Log2FC	HREC CpE (1.5h) P_{ADJ}
RND1	4.6	1.5×10^{-46}	4.4	8.9×10^{-264}	5.4	1.5×10^{-31}	-3.7	9.3×10^{-96}
RASSF10	2.6	3.3×10^{-6}	3.6	4.4×10^{-19}	7.1	5.7×10^{-10}	-1.9	6.5×10^{-5}
HEY2	3.3	7.3×10^{-6}	3.5	3.1×10^{-30}	5.3	2.1×10^{-21}	-0.9	3.7×10^{-5}
EFNB2	2.5	1.7×10^{-56}	2.6	$< 8.9 \times 10^{-264}$	2.3	1.3×10^{-70}	-1.6	2.7×10^{-73}
PRICKLE2	2.3	6.4×10^{-48}	2.6	5.7×10^{-206}	1.5	1.2×10^{-19}	-1.1	2.6×10^{-9}
SLC46A3	2.9	7.2×10^{-12}	2.6	$< 8.9 \times 10^{-264}$	1.9	3.6×10^{-16}	-2.0	3.2×10^{-14}
DLL4	2.7	7.1×10^{-54}	2.2	$< 8.9 \times 10^{-264}$	3.5	3.0×10^{-12}	-4.2	4.2×10^{-11}
RAPGEF5	1.6	1.3×10^{-51}	1.8	$< 8.9 \times 10^{-264}$	1.3	9.8×10^{-31}	-0.9	9.3×10^{-24}
PLPP3	1.6	1.3×10^{-51}	1.7	$< 8.9 \times 10^{-264}$	2.3	1.4×10^{-43}	-0.6	2.1×10^{-5}
HEY1	1.6	7.5×10^{-19}	1.6	7.4×10^{-52}	6.1	3.3×10^{-72}	-1.7	2.9×10^{-3}
LRRC32	1.0	1.7×10^{-11}	1.6	8.9×10^{-264}	1.2	3.1×10^{-14}	-0.9	6.6×10^{-7}
SAT1	2.1	9.6×10^{-38}	1.5	8.9×10^{-264}	1.6	2.9×10^{-23}	-1.1	3.6×10^{-16}
HES4	1.5	2.2×10^{-9}	1.5	1.5×10^{-166}	2.7	1.5×10^{-77}	-1.8	5.1×10^{-44}
ANKRD33B	1.4	1.3×10^{-16}	1.5	1.2×10^{-114}	1.1	2.9×10^{-12}	-0.8	1.2×10^{-7}
FLT1	1.4	6.7×10^{-5}	1.4	$< 8.9 \times 10^{-264}$	1.7	1.5×10^{-53}	-0.6	3.1×10^{-8}

Figure S3

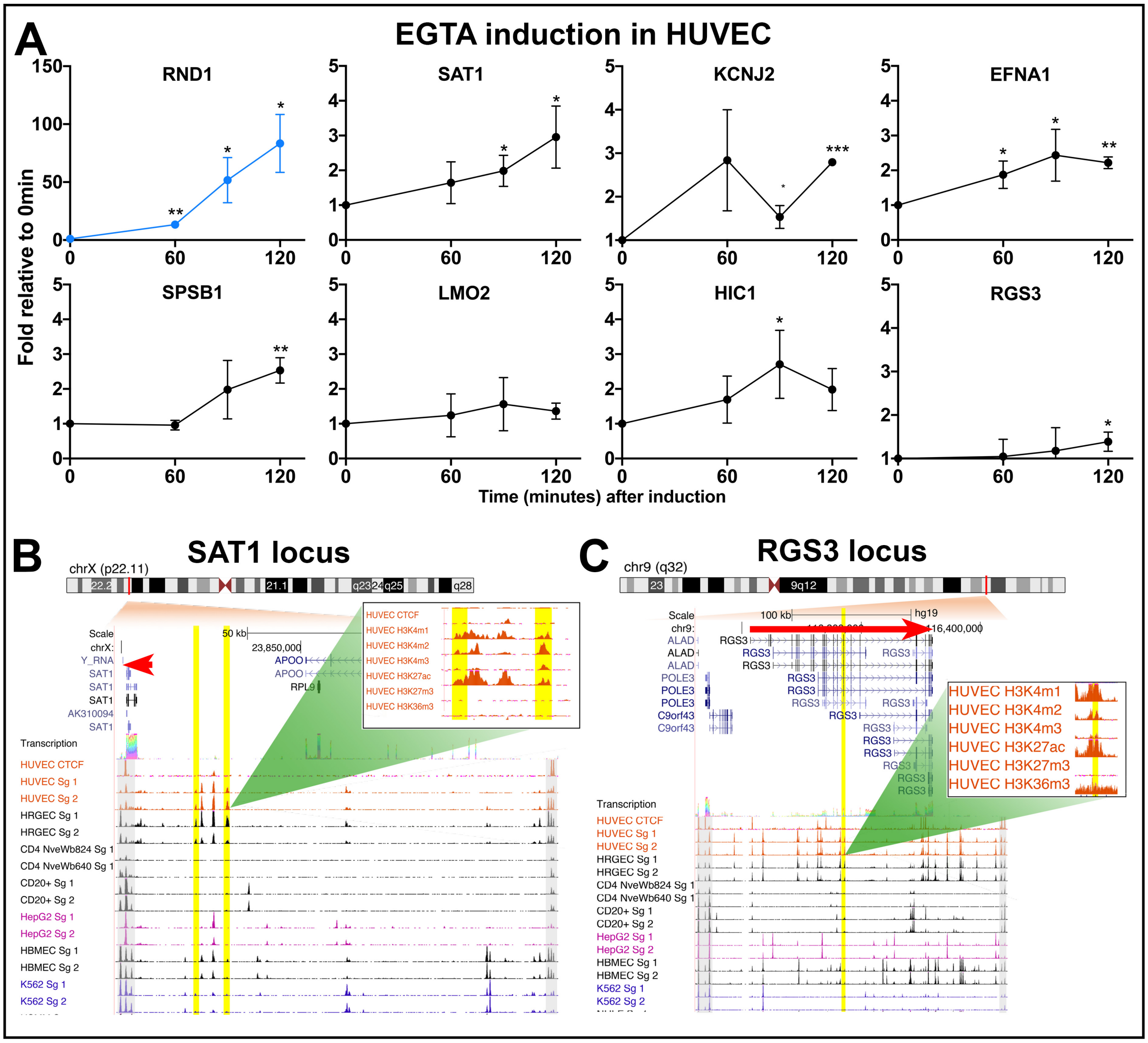

A
EGTA induction in HUVEC
RND1
SAT1
KCNJ2
EFNA1
SPSB1
LMO2
HIC1
RGS3
Fold relative to 0min
Time (minutes) after induction
B
SAT1 locus
chrX (p22.11)
C
RGS3 locus
chr9 (q32)
HUVEC H3K4m1
HUVEC H3K4m2
HUVEC H3K4m3
HUVEC H3K27ac
HUVEC H3K27m3
HUVEC H3K36m3
Transcription
HUVEC CTCF
HUVEC Sg 1
HUVEC Sg 2
HRGEC Sg 1
HRGEC Sg 2
CD4 NveWb824 Sg 1
CD4 NveWb640 Sg 1
CD20+ Sg 1
CD20+ Sg 2
HepG2 Sg 1
HepG2 Sg 2
HBMEC Sg 1
HBMEC Sg 2
K562 Sg 1
K562 Sg 2

Figure S4

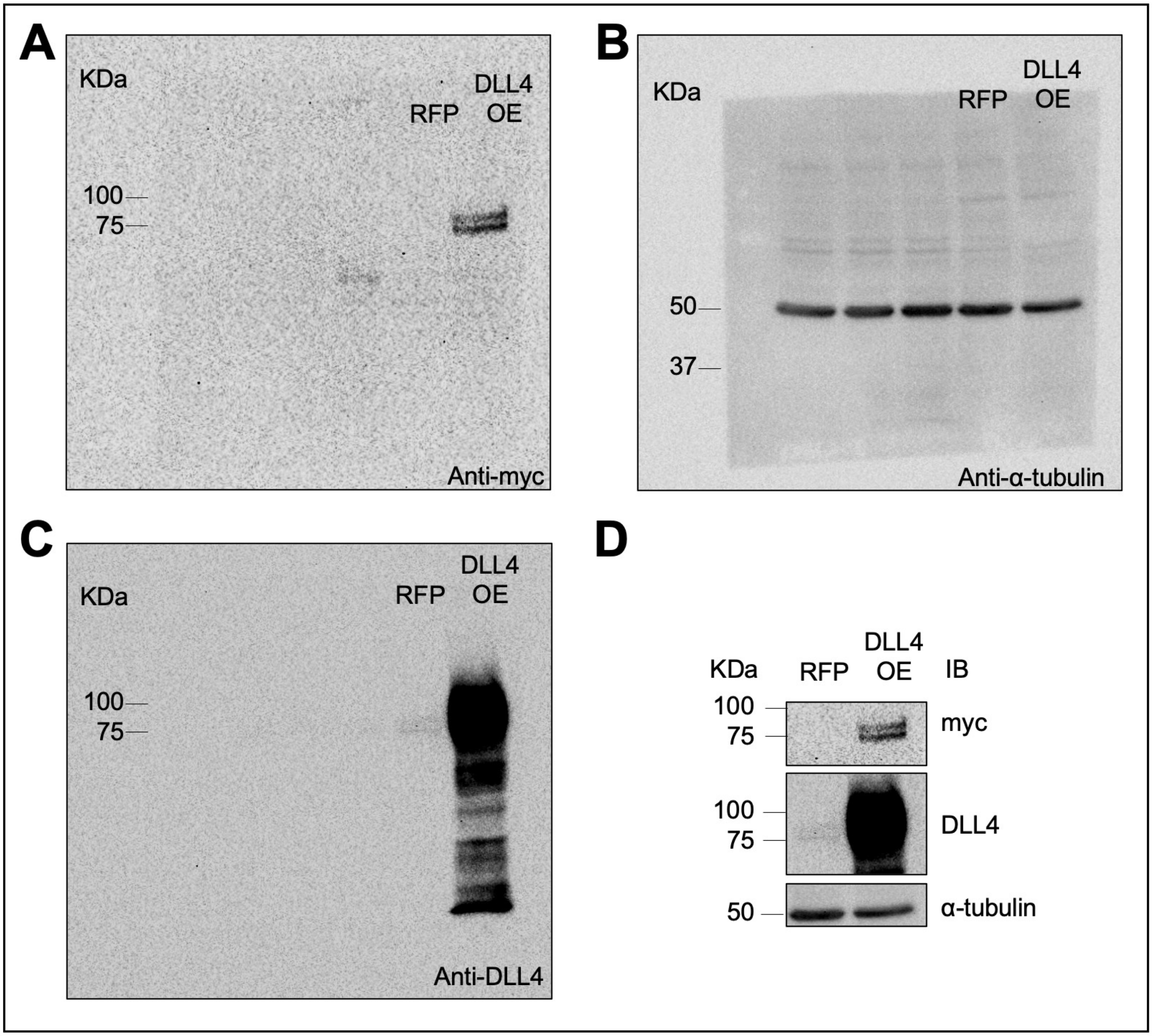
A
KDa
RFP
DLL4
OE
100
75
Anti-myc
B
KDa
RFP
DLL4
OE
50
37
Anti-α-tubulin
C
KDa
RFP
DLL4
OE
100
75
Anti-DLL4
D
KDa
RFP
DLL4
OE
IB
100
75
myc
100
75
DLL4
50
α-tubulin

Table S1

Target_gene	Primer_direction	Sequence(5'-3')
hACTB	FP	CGAGGCCCAGAGCAAGAGAG
hACTB	RP	CTCGTAGATGGGCACAGTGTG
hHEY1	FP	ATCTGCTAAGCTAGAAAAAGCCG
hHEY1	RP	GTGCGCGTCAAAGTAACCT
hHEY2	FP	GCCCGCCCTTGTCAGTATC
hHEY2	RP	CCAGGGTCGGTAAGGTTTATTG
hHES1	FP	CCTGTCATCCCCGTCTACAC
hHES1	RP	CACATGGAGTCCGCCGTAA
hNRARP	FP	TCAACGTGAACTCGTTCGGG
hNRARP	RP	ACTTCGCCTTGGTGATGAGAT
hRND1	FP	CTATCCAGAGACCTATGTGCC
hRND1	RP	CGGACATTATCGTAGTAGGGAG
hSAT1	FP	ACCCGTGGATTGGCAAGTTAT
hSAT1	RP	TGCAACCTGGCTTAGATTCTTC
hLMO2	FP	AAGCGGATTCGTGCCTATGAG
hLMO2	RP	AGTTGATGAGGAGGTATCTGTCA
hHIC1	FP	GTCGTGCGACAAGAGCTACAA
hHIC1	RP	CGTTGCTGTGCGAACTTGC
hRGS3	FP	ACCTACCTGCTGGTCAAGAAC
hRGS3	RP	GGGTGGGGGATTCCTGGAT
hUNC5B	FP	CTGGGACCTTATGCCTTCAA
hUNC5B	RP	CGCTTTGGTGGCAAAGTAAT
hKCNJ2	FP	TTCAGTCACAATGCCGTGATT
hKCNJ2	RP	GCTTTTCCGAAGATTGCCCA
hSPSB1	FP	CCGGCTGGATCTGCTACTG
hSPSB1	RP	TCGGTCGTTGTTGTTCCATGA
hEFNA1	FP	TCAGGCCCATGACAATCCAC
hEFNA1	RP	GTGACCGATGCTATGTAGAACC
hRND1_Enhancer_ChIP	FP	GCAGGGATAGACTTGCCTTT
hRND1_Enhancer_ChIP	RP	TCTGAGTCTCTGGGTTCCTTAT

SUPPLEMENTARY FIGURES:

Figure S1: Validation of HUVEC and HREC endothelial lines.

A. To ensure that endothelial cells retained endothelial identity over time in culture, we examined expression of critical endothelial-specific markers CD31 (green) and VE-cadherin (red) at passages beyond those used for experiments. Representative batch (U2) of HUVEC isolated from an umbilical cord by the Kitajewski lab and used at passages 4-5 retains CD31 and VE-cadherin expression at passage 6.

B. Commercially isolated HUVEC from Lonza at passage 6 show similar retention of CD31 and VE-cadherin expression.

C. Commercially isolated HREC from Cell Systems, used for experiments at passage 6-8, retained expression of CD31 and VE-cadherin at passage 14.

Figure S2: Analysis of RNAseq data to determine novel Notch targets.

A. Principal component analysis (PCA) plot of RNA-seq data from DLL4 TLA in HUVEC. PC1 segregation shows differences between individual donors, while PC2 shows differences between Dll4-induced and control samples. Donor differences were controlled used the batch effect function in DESeq2.

B. PCA plot of RNA-seq data from DLL4 TLA in HREC, from a single donor.

C. PCA plot of RNA-seq data from EGTA induction in HREC prior to comparing to CpE inhibition.

D. PCA plot of RNAseq data from HREC induced with EGTA and inhibited with CpE.

E. The 20 genes whose induction by EGTA was most strongly repressed by CpE treatment, represented as fold inhibition by CpE.

F. GO pathways significantly enriched in the 52 genes significantly regulated across all tested *in vitro* conditions.

G. Table of 15 genes most highly regulated across all tested *in vitro* conditions, sorted by fold change, which include previously identified (purple) and novel (white) Notch targets.

Figure S3: Validation of Notch-responsiveness of novel consensus targets.

A. All 8 novel endothelial Notch targets show significant induction in response to EGTA in HUVEC as well as HREC (Figure 4), and RND1 is consistently the most strongly induced. The y axes are scaled for each gene to visualize induction of different magnitudes.

B. ENCODE visualization of endothelial-specific DHS peaks in the *SAT1* locus displaying active histone marks, suggestive of an endothelial-specific enhancer region. Some DNAse hypersensitivity peaks appear only in endothelial cell types (yellow bar) and show histone methylation patterns consistent with active enhancers (insert box), suggesting that these peaks are endothelial-specific enhancers. Gray bars mark CTCF-binding insulator regions.

C. Similar analysis of the *RGS3* locus.

Figure S4: Uncropped Western Blots.

Uncropped blots of anti-myc (A), anti-tubulin (B), anti-DLL4, and assembled panel for Figure 4A.

Table S1: Primer sequences

Table containing the forward (FP) and reverse (RP) primer sequences for the genes listed. All sequences are in the 5’-3’ direction.
